# Association between different peritoneal dialysis catheter placement methods and short‐term postoperative complications

**DOI:** 10.1186/s12882-021-02340-y

**Published:** 2021-04-26

**Authors:** Yibo Ma, Shuiqing Liu, Min Yang, Yun Zou, Dong Xue, Yanping Liu, Yufeng Wang, Xiao Xie, Hui Chen

**Affiliations:** 1 Department of Ultrasound, the Third Affiliated Hospital of Soochow University, 213003 Changzhou, China; 2 Department of Nephrology, the Third Affiliated Hospital of Soochow University, 213003 Changzhou, China; 3 Department of Urology, the Third Affiliated Hospital of Soochow University, 213003 Changzhou, China

**Keywords:** Peritoneal dialysis, Catheter placement, Complication, Catheter migration

## Abstract

**Background:**

Considering that current peritoneal dialysis has its own shortcomings, In this study, the Seldinger technique was modified to explore the relationship between different catheter placement methods of peritoneal dialysis and short-term postoperative complications.

**Methods:**

We retrospectively analyzed the data of 157 patients who received peritoneal dialysis in the Department of Nephrology of our hospital from January 2017 to December 2019. According to different catheter placement methods, the patients were divided into three groups: 111 cases of open surgery technique, 23 cases of Seldinger technique, and 23 cases of modified Seldinger technique (ultrasound-guided Veress needle puncture). The general data, laboratory indexes, and abdominal infection and catheter-related complications within one month postoperatively were collected.

**Results:**

There were 48 (31.0 %) cases of complications in 157 patients within one month postoperatively, which were mainly catheter-related complications (45 cases, 29.0 %). The incidence of catheter tip peritoneal drift (catheter migration) in the three groups was 27.3 %, 39.1 %, and 9.1 %, respectively, with no significant difference between groups (*P* = 0.069). Univariate logistic regression analysis showed that the systolic blood pressure, history of abdominal and pelvic surgery, creatinine, and modified Seldinger technique were possible impact factors of catheter migration (*P* < 0.10). After fully adjusting for confounding factors, Compared with the open surgery group, the modified Seldinger method group significantly reduced the risk of catheter migration with an OR of 0.161 (95 % confidence interval: 0.027–0.961, *P* = 0.045); However, the difference between the Seldinger method group and the open surgery group was not significant, with an OR of 1.061 (95 % confidence interval: 0.308–3.649, *P* = 0.926). Curve fitting showed that the average incidence of catheter migration in the three groups was 27.3 % (95% CI: 15.9-42.7 %), 28.5 % (95% CI: 10.7-56.9 %), and 5.7 % (95% CI: 1.0-27.0 %); the modified Seldinger method has the lowest average incidence of catheter migration.

**Conclusions:**

Modified Seldinger technique can significantly reduce catheter-related short-term complications after peritoneal dialysis, and it is especially effective in reducing the incidence of catheter migration. Modified Seldinger technique is a safe and feasible method for the placement of a peritoneal dialysis catheter.

## Background

Dialysis is one of the main treatment methods for end-stage renal disease (ESRD), including hemodialysis (HD) and peritoneal dialysis (PD). Compared with HD, PD has the advantages of wide application range, protection of residual renal function, can be done at home, operate easily and safely [1–4]. Therefore, it has been widely used in clinical practice. The therapeutic effect of PD mainly depends on the successful placement of PD catheters, which can reduce complications such as peritonitis and catheter migration and prolong patients’ lives [5, 6].

At present, PD catheter placement methods mainly include traditional open surgery, laparoscopic surgery, and percutaneous needle guidewire technique (Seldinger technique) [7, 8]. Open surgery is widely used in clinical practice. Open surgery catheter placement technique is simple, but postoperative complications are high [9–11]. Peritoneal dialysis catheter displacement is the most common complication of open surgery peritoneal dialysis catheter placement, with an incidence of 10-20 % [10, 12]. Laparoscopic catheter placement needs general anesthesia, the requirements for equipment and operation techniques are relatively high, and the surgery cost is high. Therefore, the application of laparoscopic technique in peritoneal dialysis catheter placement is limited in China. Recent studies have shown that [13, 14], catheter-related complications and catheter survival rate of Seldinger technique are similar to those of laparoscopic catheter placement. Seldinger technique is an effective and safe catheter placement technique [15]. Also, Seldinger technique has the advantages of less traumatic, less intraoperative pain, and less economic burden, and the operation technique is easy for nephrologists to master. However, whether it can reduce postoperative complications than traditional open surgery methods remain controversial [16]. At present, there are few relevant studies. Some researchers improved Seldinger technique using ultrasound-guided technology (modified Seldinger technique), which is comparable to laparoscopic catheter placement and can improve catheter placement’s success rate [17]. This study retrospectively analyzed the clinical data and short-term complications of patients with different catheter placement methods, including open surgery, Seldinger technique, and modified Seldinger technique, to explore the correlation between different catheter placement methods and short-term postoperative complications.

## Methods

**Fig. 1:**
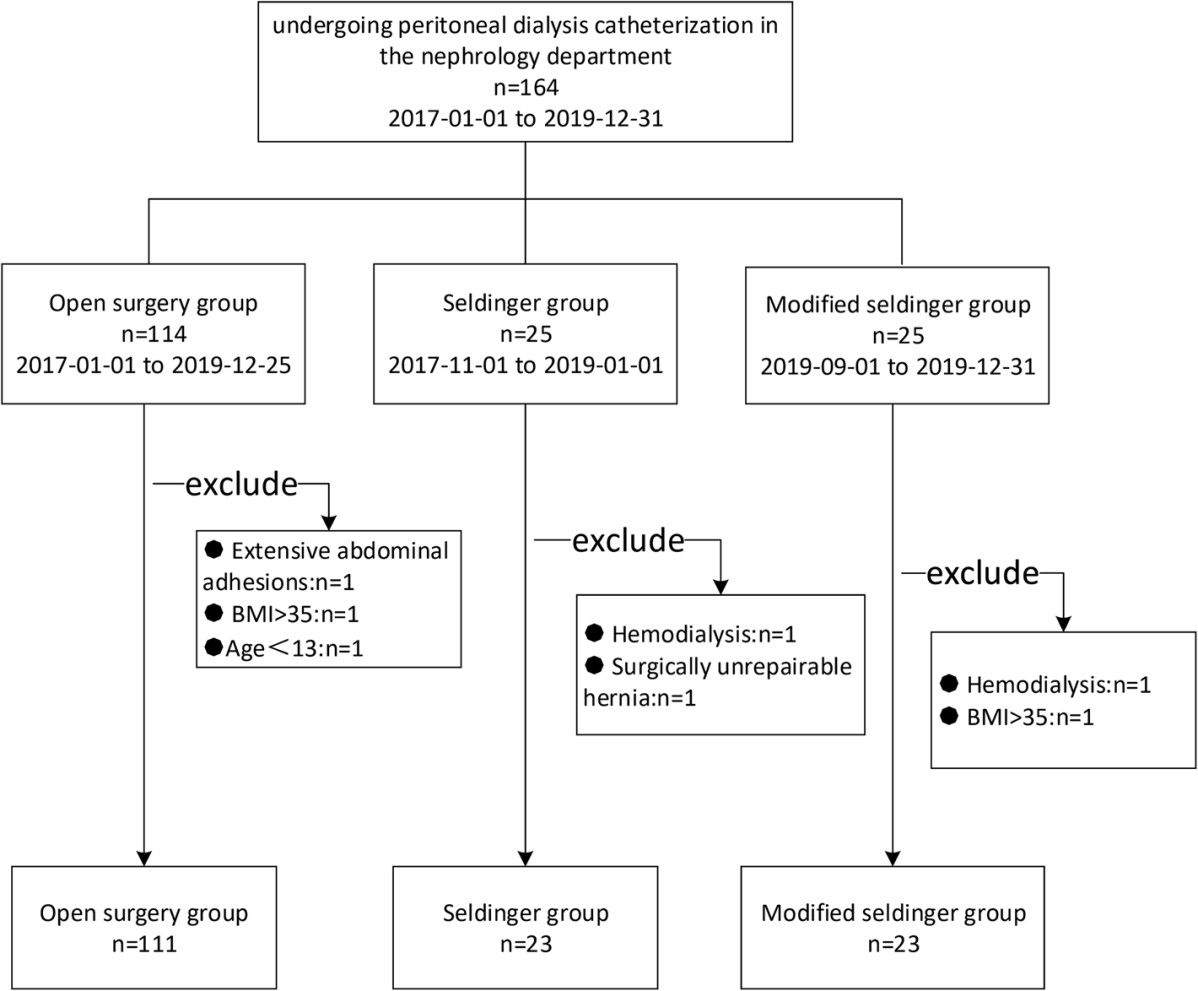
Flow chart of patient selection

This was a retrospective cohort study. We analyzed the data of patients who underwent peritoneal dialysis catheter placement for the first time from January 2017 to December 2019 in the Department of Nephrology, the Third Affiliated Hospital of Soochow University. This study was approved by the Institutional Ethics Committee of our hospital for retrospective analysis (ethics number: 2020-WD-027), and each patient signed the informed consent form before surgery. Patients inclusion criteria: age ≥ 13 years old; patients with acute renal failure or chronic renal failure who need long-term renal replacement therapy; patients with unstable hemodynamics, coagulation dysfunction, bleeding tendency, and difficulty in establishing vascular access. Patients exclusion criteria: hemodialysis patients; patients with severe, extensive abdominal adhesion; hernias cannot be repaired by surgery; patients with body mass index (BMI) > 35; patients with severe mental disorders without appropriate accompanying management. The patient selection process is shown in Fig. 1.

The general information, past medical history, laboratory indexes, duration of catheter placement operation, and short-term complications (including abdominal infection and catheter-related complications, such as catheter migration, pericatheter leaks, catheter plugging, and tunnel infection) were collected.

### Ultrasonic instruments and diagnostic criteria

The thickness of subcutaneous adipose layer and muscular layer of abdominal wall, the position of the iliac artery in abdominal wall, the diameter of blood vessels in subcutaneous tissue, and the relative distance between visceral movement and abdominal wall movement were measured by ultrasonic diagnostic instrument (Mindray M8 Super, China) with linear array probe (frequency 4–12 MHz).

### Peritoneal dialysis catheter placement

The three catheter placement methods are all performed by a nephrologist. An experienced doctor performs the open surgery technique, and two Specific doctors use the Seldinger or modified Seldinger technique. All three groups of patients used straight tubes, and the tubes were placed in a curved path at an angle of 45°. Patients in each group started peritoneal dialysis on the third day after catheterization [18, 19], the peritoneal dialysis fluid is gradually increased from a small dose (800ml), and using normal dose peritoneal dialysis fluid one week later. The specific tube placement method is as follows:

The traditional open surgery method was operated as follows: a 3–5 cm incision was cut on the skin and subcutaneous tissue under local infiltration anesthesia, the anterior sheath of rectus abdominis was cut longitudinally, and the rectus abdominis was dissociated bluntly to expose the posterior sheath of rectus abdominis. A small incision was cut in the posterior sheath, and the peritoneal dialysis catheter was placed in the abdominal cavity, uterus, and rectal lacuna under the guidance of guidewire [7, 20]. Seldinger technique was to use the Tenckhoff trocar, guidewire, and sheath system for operation. The catheter was inserted into the abdominal cavity without direct vision, and the deep polyester sheath was placed only outside the abdominal muscle tissue [21]. The modified Seldinger technique was as follows: under the guidance of ultrasound, the Veress needle enters the abdominal cavity from the anterior sheath of rectus abdominis. Normal saline was injected to ensure that there was no obstruction. The guidewire was placed in the peritoneal fluid above the bladder. The Veress needle was removed, and a dilator was placed along the guidewire to dilate the anterior sheath of rectus abdominis until the peritoneum. Normal saline was injected again to ensure a smooth flow. The avulsion sheath with the core was inserted along the guidewire, and the peritoneal dialysis catheter was placed along the avulsion sheath. The sheath was torn from both sides until all parts lower than the polyester sheath in the peritoneal dialysis catheter entered. The peritoneal fluid drainage was unobstructed [17, 22, 23].

### Examination of catheter function and diagnosis of catheter migration

Inject 200ml of peritoneal dialysis fluid during the operation. If it is injected and discharged smoothly and can be discharged completely, it indicates that the catheter functions well. All patients were diagnosed by X-ray radiography the next day after catheter placement to determine that the catheter position was normal. After the operation, if the peritoneal dialysis fluid is put into the catheter smoothly, and the discharge is not smooth, catheter migration is suspected clinically, and X-ray reexamination is performed to diagnose catheter migration.

### Statistical analysis

The statistical data of all patients grouped by different catheterization methods were expressed as the frequency and proportion for categorical variables and the mean ± SD for continuous variables. Chi-square test (categorical variable), one-way ANOVA (normal distribution continuous variable), and Kruskal-Wallis test (partial continuous variable) were used to analyze the differences between groups.

Univariate logistic regression analysis was used to calculate the correlation between different clinical features and short-term postoperative complications. The generalized linear models with a logit link were used to test the independent and comprehensive impacts of catheter placement methods on short-term complications (catheter migration). We calculated unadjusted and adjusted estimates using exact methods and asymptotic methods, respectively. Covariates were included as potential confounders in the final models if they changed the estimates of catheter placement method on catheter migration by more than 10 % or were significantly associated with catheter migration (*P* < 0.10). Odds ratio (OR) and 95 % confidence interval were calculated. The open surgery method was used as a reference for catheter placement methods.

The generalized additive models (GAM) were used to test the correlation between the catheter placement method and the occurrence of catheter migration, and a broken line diagram was drawn accordingly. All data were analyzed using R software (version 3.4.3, http://www.R-project.org). *P* < 0.05 was considered statistically significant.

## Results

### 1. Comparison of the general data

Finally, 157 patients were enrolled in this study, including 90 males and 67 females, with an average age of 47.1 ± 15.1 years (range: 13–84 years). The patients were divided into three groups according to the catheter placement method: 111 cases in open surgery group, 23 cases in Seldinger technique group, and 23 cases in modified Seldinger technique group. A comparison of the general data of the three groups is shown in Table 1. The differences in the history of hypertension, history of abdominal and pelvic surgery, and duration of catheter placement procedure were statistically significant (*P* < 0.05). In this study, 48 cases (31.0 %) had complications within one month after the procedure, including 5 cases of abdominal infection (3.2 %) and 45 cases (29.0 %) of catheter-related complications. Two cases had both abdominal infection and catheter-related complications. Among catheter-related complications, catheter tip abdominal migration incidence in the three groups was 27.3 %, 39.1 %, and 9.1 %, respectively, with no significant difference between each other (*P* = 0.069).

**Table 1:** General information of patients with different catheter placement methods

catheter placement method	Total	Open surgery	Seldinger technique	Modified Seldinger technique	*P*-value
N	157	111	23	23	
Age(years)	47.1 ± 15.1	47.6 ± 15.1	46.5 ± 17.2	45.3 ± 13.1	0.788
Gender					0.654
Female	67 (42.7 %)	48 (43.2 %)	11 (47.8 %)	8 (34.8 %)	
Male	90 (57.3 %)	63 (56.8 %)	12 (52.2 %)	15 (65.2 %)	
BMI (kg/m2)	22.2 ± 3.2	22.2 ± 3.1	22.6 ± 4.3	21.4 ± 2.5	0.456
Systolic pressure(mmHg)	157.4 ± 25.3	155.9 ± 24.6	159.5 ± 27.8	162.4 ± 26.0	0.490
Diastolic pressure (mmHg)	91.0 ± 16.0	89.6 ± 15.2	92.8 ± 18.3	95.6 ± 17.2	0.223
History of diabetes mellitus	22 (14.0 %)	12 (10.8 %)	4 (17.4 %)	6 (26.1 %)	0.139
History of hypertension	142 (90.4 %)	101 (91.0 %)	23 (100.0 %)	18 (78.3 %)	0.040
History of glomerulonephritis	107 (68.2 %)	72 (64.9 %)	18 (78.3 %)	17 (73.9 %)	0.370
History of ischemic heart disease	16 (10.2 %)	8 (7.2 %)	3 (13.0 %)	5 (21.7 %)	0.098
History of abdominal and pelvic surgery	32 (20.4 %)	22 (19.8 %)	1 (4.3 %)	9 (39.1 %)	0.013
Laboratory indexes					
Hemoglobin (g/L)	82.8 ± 14.9	83.8 ± 14.6	81.3 ± 12.6	79.5 ± 18.4	0.403
Albumin (g/L)	34.6 ± 4.3	35.0 ± 4.1	33.3 ± 4.3	34.4 ± 5.4	0.215
Urea nitrogen (mmol/L)	30.2 ± 10.2	29.7 ± 9.8	29.6 ± 12.7	33.2 ± 9.7	0.306
Creatinine (µmol/L)	864.4 ± 284.3	850.2 ± 265.8	879.2 ± 329.6	918.4 ± 327.1	0.560
Duration of catheter placement procedure (min)	83.7 ± 30.6	95.0 ± 28.6	51.0 ± 11.7	62.0 ± 12.8	< 0.001
Complications within 1 month	48 (31.0 %)	32 (29.1 %)	12 (52.2 %)	4 (18.2 %)	0.035
Abdominal infection	5 (3.2 %)	3 (2.7 %)	1 (4.3 %)	1 (4.5 %)	0.859
Catheter-related complications	45 (29.0 %)	31 (28.2 %)	11 (47.8 %)	3 (13.6 %)	0.039
Catheter migration	41 (26.5 %)	30 (27.3 %)	9 (39.1 %)	2 (9.1 %)	0.069
Pericatheter leaks	6 (3.9 %)	4 (3.6 %)	2 (8.7 %)	0 (0.0 %)	0.310
Catheter blockage	4 (2.6 %)	3 (2.7 %)	0 (0.0 %)	1 (4.5 %)	0.620
Tunnel infection	3 (1.9 %)	1 (0.9 %)	1 (4.3 %)	1 (4.5 %)	0.354

Data in the Table: Mean ± SD / N (%)

### 2. Crude association of clinical features of the patients and catheter migration

The incidence of catheter migration within one month postoperatively was used as the dependent variable (Y = 1). After adjusting age, gender, 14 clinical features, including catheter placement method, were used as independent variables for univariate logistic regression analysis. The results showed that systolic blood pressure, history of abdominal and pelvic surgery, creatinine, and modified Seldinger technique were the possible impact factors of catheter migration (*P* < 0.10). The specific data are shown in Table 2.

**Table 2:** Crude association of clinical features of the patients and catheter migration

Clinical feature	OR (95 %CI)	*P*-value
BMI (kg/m^2^)	1.019 (0.912, 1.140)	0.737
Systolic pressure(mmHg)	1.013 (0.998, 1.027)	0.087
Diastolic pressure(mmHg)	1.021 (0.996, 1.046)	0.100
History of diabetes	2.078 (0.690, 6.263)	0.194
History of hypertension	2.152 (0.450, 10.296)	0.337
History of glomerulonephritis	0.783 (0.345, 1.778)	0.559
History of ischemic heart disease	0.907 (0.275, 2.996)	0.873
History of abdominal and pelvic surgery	0.369 (0.126, 1.080)	0.069
Hemoglobin (g/L)	0.985 (0.961, 1.010)	0.236
Albumin (g/L)	0.981 (0.898, 1.072)	0.675
Urea nitrogen (mmol/L)	1.030 (0.994, 1.066)	0.101
Creatinine (µmol/L)	1.001 (1.000, 1.002)	0.069
Duration of catheter placement procedure (min)	0.997 (0.985, 1.009)	0.610
Catheter placement method		
Open surgery	1.0	
Seldinger technique	1.715 (0.669, 4.396)	0.262
Modified Seldinger technique	0.276 (0.061, 1.257)	0.096

Data in the table: OR (95 % CI) *P*-value;

Adjustment variables: gender, age

### 3. Multivariate regression for the effect of catheter placement method on catheter migration

Table 3 shows the results of univariate and multivariate logistic regression analysis, which were used for different catheter placement methods. Unadjusted covariates were equivalent to univariate logistic regression analysis (only adjusted for gender, age). Primarily adjusted covariates included age, systolic blood pressure, history of abdominal and pelvic surgery, creatinine. Fully adjusted covariates included gender, age, BMI, systolic blood pressure, diastolic blood pressure, diabetes, hypertension, history of glomerulonephritis, history of ischemic heart disease, history of abdominal and pelvic surgery, hemoglobin, urea nitrogen, creatinine, duration of catheter placement procedure (min).

**Table 3:** Multivariate regression for the effect of catheter placement method on catheter migration

Exposure	Non-adjusted		Adjust I		Adjust II	
Catheter placement method	OR (95 %CI)	*P*-value	OR (95 %CI)	*P*-value	OR (95 %CI)	*P*-value
Open surgery	1.0		1.0		1.0	
Seldinger technique	1.715 (0.669, 4.396)	0.262	1.456 (0.543, 3.906)	0.456	1.061 (0.308, 3.649)	0.926
Modified Seldinger technique	0.276 (0.061, 1.257)	0.096	0.247 (0.051, 1.198)	0.083	0.161 (0.027, 0.961)	0.045

Data in the table: OR (95 % CI) *P*-value;

Non-adjusted model adjusts for: gender, age;

Adjust I model adjust for: gender, age, systolic pressure, history of abdominal and pelvic surgery, creatinine;

Adjust II model adjust for: gender, age, BMI, systolic blood pressure, diastolic blood pressure, diabetes, hypertension, history of glomerulonephritis, history of ischemic heart disease, history of abdominal and pelvic surgery, hemoglobin, urea nitrogen, creatinine, duration of catheter placement procedure (min)

In the regression equation with unadjusted and initially adjusted covariates, the difference between the modified Seldinger group and the open surgery group was not significant (*P* = 0.096 and 0.083). In the regression equation with fully adjusted covariates, the risk of catheter migration in the modified Seldinger group was significantly lower than that in the open surgery group, with an OR of 0.161 (95 %CI: 0.027–0.961, *P* = 0.045). In the three regression equations, the difference between the Seldinger method group and the open surgery group was not significant (all *P* > 0.05).

### 4. Curve fitting

**Fig. 2:**
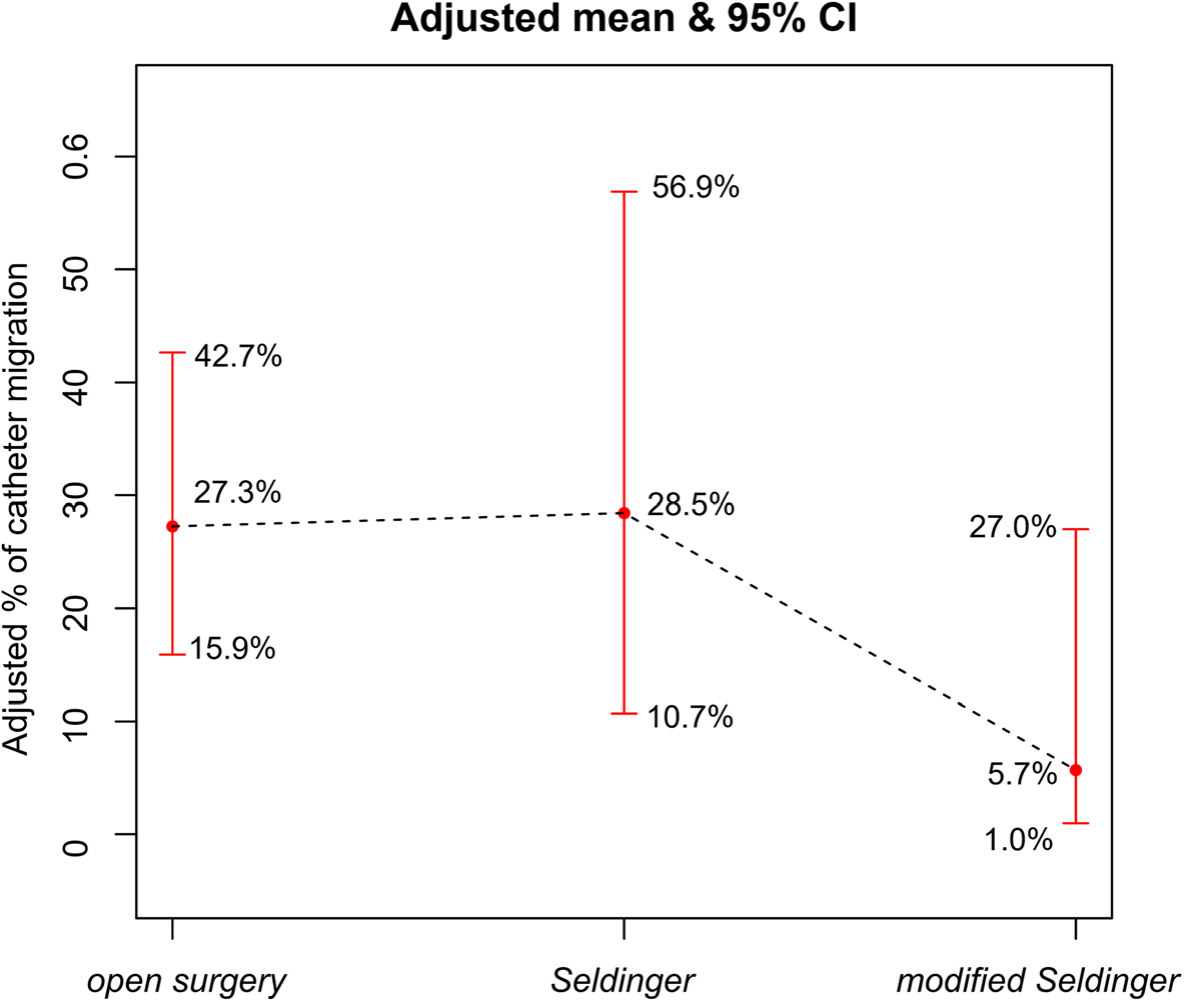
The impact of different catheter placement methods on catheter migration incidence (the black dotted line indicates the fitted line of the incidence of catheter migration and catheter placement method; the red line is the 95 % confidence interval). Adjust for: gender, age, BMI, systolic blood pressure, diastolic blood pressure, diabetes, hypertension, history of glomerulonephritis, history of ischemic heart disease, history of abdominal and pelvic surgery, hemoglobin, urea nitrogen, creatinine, duration of catheter placement procedure (min)

GAM was used to examine the correlation between the catheter placement method and catheter migration. The results showed that after adjusting for gender, age, BMI, systolic blood pressure, diastolic blood pressure, diabetes, hypertension, history of glomerulonephritis, history of ischemic heart disease, history of abdominal and pelvic surgery, hemoglobin, urea nitrogen, creatinine, duration of catheter placement procedure (min), the average incidence of catheter migration in the three groups were 27.3 % (95 %CI: 15.9-42.7 %), 28.5 % (95 %CI: 10.7-56.9 %) and 5.7 % (95 %CI: 1.0-27.0 %), the modified Seldinger method has the lowest average incidence (see Fig. 2).

## Discussion

The traditional open surgical method of catheter placement is to make a small incision in the abdominal wall, and the visual field of observation of the abdominal cavity is very limited. Moreover, blind insertion can cause bleeding of blood vessels under the abdominal wall and serious complications such as injuring the intestine and bladder in the abdominal cavity. Compared with the traditional open surgery method, Seldinger technique is relatively easy to master for nephrologists, and it can improve the utilization of peritoneal dialysis [24]. However, compared with the traditional open surgical method, whether Seldinger’s method can reduce postoperative complications is still controversial [14, 25]. A meta-analysis of different catheter placement methods in peritoneal dialysis (PD) by Xie et al. [16] showed that the one-year dysfunction-free catheter survival, one-year dysfunction-and-infection-free catheter survival, and overall catheter survival were similar between the traditional open surgery method and Seldinger technique. Lee et al. showed that compared with open surgery method, Seldinger technique could reduce catheter-related complications [26, 27]. Same as the traditional open surgery method, the Seldinger method is also a blind puncture, which may cause the risk of intestinal perforation and the risk of early mechanical complications, which limits its application in China [28]. To overcome the Seldinger method’s deficiencies and reduce surgery-related risk, we use ultrasound-guided puncture to overcome the shortcomings of blind puncture. The short-term complications of different catheter placement methods were compared and found that the modified Seldinger method could effectively reduce the incidence of Catheter migration within one month after surgery.

There were significant differences in the history of hypertension, history of abdominal and pelvic surgery and duration of catheter placement procedure among patients with different catheter placement methods. Because the history of abdominal surgery is the relative contraindication of PD, in this study, the patients with a history of abdominal and pelvic surgery in Seldinger group were significantly less than those in the other two groups. However, the history of abdominal and pelvic surgery is no longer a contraindication for the modified Seldinger technique, and therefore patients with a history of abdominal and pelvic surgery are more suitable for Seldinger technique. Consistent with the results of Peng et al. [5], we found that Seldinger technique can significantly shorten the operation time compared with the open surgery method. Consistent with the results of Medani et al. [10], in this study, the modified Seldinger technique can significantly reduce catheter-related complications within one month postoperatively, especially the occurrence of catheter migration, compared with open surgery method. It has been reported that the incidence of catheter migration after peritoneal dialysis is 5-35 %, of which 85.9 % occurs in the first two weeks after catheter insertion [29, 30]. Our results showed that the incidence of catheter migration in modified Seldinger group was 9.1 %, which was significantly lower than that of the other two groups, but there was no significant difference among the three groups (*P* = 0.069). We speculated that there might be confounding factors.

Univariate analysis showed that the history of abdominal and pelvic surgery, Systolic blood pressure, creatinine, and modified Seldinger technique might associate with the occurrence of catheter migration. As is well-known, patients with a history of abdominal and pelvic surgery often have abdominal adhesions, which will increase the surgery-related mechanical complications, and thus is the relative contraindication of PD [31]. In this study, the correlation between the history of abdominal and pelvic surgery and catheter migration is contrary to previous studies. We found that the higher the history of abdominal and pelvic surgery, the lower the occurrence of catheter migration. This result may be since patients with a history of surgery tend to choose modified Seldinger technique. Thus, the history of abdominal and pelvic surgery is a confounding factor. Our results showed that systolic blood pressure might affect the occurrence of catheter migration, Combined with the differences in the history of hypertension in different groups. Thus, we consider that blood pressure may also be a confounding factor. Moreover, we found that creatinine level was also correlated with catheter migration, and the incidence of catheter migration increased with the elevation of creatinine level. Crabtree et al. [20] considered that excessive bending of the catheter in the subcutaneous track to produce a lateral or downward exit site direction, and poor fixation of the transmural segment is the basis of catheter migration. According to this point of view, we deduce that uremia may affect wound healing, affecting the fixation of the transmural segment of the catheter, resulting in an increase in the incidence of catheter migration.

After fully adjusting the confounding factors, the modified Seldinger technique significantly reduced the occurrence of catheter migration, and the curve fitting showed the same result. In agreement with our results, Shanmugalingam et al. [32] found that preoperative abdominal ultrasound evaluation can effectively screen qualified patients for percutaneous PD catheter placement and significantly reduce the risk of catheter placement associated with mechanical complications. An important factor contributing to postoperative catheter migration is the inaccurate placement of the catheter tip into the uterine, rectum, or bladder. Ultrasound guidance can overcome the shortcoming of previous blind puncture and ensure the accurate positioning of PD catheters, which may be an important factor for the low incidence of postoperative catheter migration. Ultrasound-guided percutaneous puncture has been reported previously. Studies [32, 33] have shown that ultrasound can be used as a useful diagnostic tool to evaluate the catheter position of patients with peritoneal dialysis catheter dysfunction and may replace traditional abdominal X-ray examination. Also, ultrasound can clearly show the position of the probe tip, identify the subcutaneous tissue, rectus abdominis, abdominal visceral organs, and other structures to avoid abdominal vascular damage [31, 34].

During the catheter insertion into the abdominal cavity, accidental injury to the intestines and bladder may occur. Previous studies have shown that the injury usually occurs when the catheter enters the abdominal cavity or when the PD catheter and the Needle core are pushed into the abdomen [7]. The Veress needle puncture can effectively prevent the puncture needle from damaging the abdominal visceral organs, but it will lose its protective effect in patients with abdominal adhesions. In this study, ultrasound-guided Veress needle puncture was used, which can effectively avoid the injury of abdominal organs even for patients with abdominal adhesion. At present, Veress needle is widely used in laparoscopic surgery [35, 36] but rarely used in peritoneal dialysis puncture. Our results suggested that an ultrasound-guided Veress needle can be safely used for peritoneal dialysis puncture, especially in patients with a previous abdominal surgery history.

The limitations of this study include the following: Firstly, this study is a single-center retrospective study. Due to the small cohort, only the catheter migration with the highest incidence of postoperative complications was selected for analysis. Besides, due to the limited number of cases of the Seldinger method and the modified Seldinger method, the statistical power may not be high enough. There are differences in the history of hypertension and the history of abdominal and pelvic surgery among the three groups, suggesting that there may be selection bias. Secondly, the average BMI of the patients in this study was low, and the results obtained may not apply to obese people. It is well known that obesity is a relative contraindication for peritoneal dialysis (PD). This is mainly due to early data showing that obese individuals have poor PD results, including increased risk of death, insufficient solute removal, higher risk of infection complications, and early technical failure [37]. Monika et al. [38] found that whether modified laparoscopy, traditional laparoscopy, or open surgery is used for catheter placement, obesity does not increase complications or shorten the survival time of non-functional PD catheters. The results of this study challenge previous ideas. Future research on peritoneal dialysis for obese people is of great significance. Lastly, since all cases came from one research center, there may be intraoperative deviations caused by the skills of the nephrologists who operated the catheter placement. Therefore, the results of this study need to be further verified by multicenter and large-scale studies.

## Conclusions

In conclusion, compared with open surgery method and Seldinger technique, modified Seldinger technique can significantly reduce the incidence of catheter migration within 1 month after surgery, which is a safe and feasible method for peritoneal dialysis catheter placement. Because it has the advantages of easy mastering of operation technology, saving operation time and economic cost, and no need for general anesthesia, it is expected to be developed as the preferred method of emergency peritoneal dialysis catheter placement in nephrology in the future.

## Data Availability

The datasets used and analysed during the current study are available from the corresponding author on reasonable request.

## Abbreviations

ESRD: End-stage renal disease
HD: Hemodialysis
PD: Peritoneal dialysis
BMI: Body mass index
OR: Odds ratio
GAM: The generalized additive models
